# Comparative evaluation of feature reduction methods for drug response prediction

**DOI:** 10.1038/s41598-024-81866-1

**Published:** 2024-12-28

**Authors:** Farzaneh Firoozbakht, Behnam Yousefi, Olga Tsoy, Jan Baumbach, Benno Schwikowski

**Affiliations:** 1 Institute for Computational Systems Biology, University of Hamburg, Hamburg, Germany; 2 Computational Systems Biomedicine Lab, Institut Pasteur, Université Paris Cité, Paris, France; 3 École Doctorale Complexite du vivant, Sorbonne Université, Paris, France; 4 Institute of Medical Systems Biology, Center for Biomedical AI (bAIome), Center for Molecular Neurobiology (ZMNH), University Medical Center Hamburg-Eppendorf, 20251 Hamburg, Germany; 5 Computational BioMedicine Lab, University of Southern Denmark, Odense, Denmark

**Keywords:** Drug response prediction, Feature reduction, Feature selection, Knowledge-based features, Machine learning, Tumour biomarkers, Machine learning, Predictive medicine, Molecular medicine

## Abstract

Personalized medicine aims to tailor medical treatments to individual patients, and predicting drug responses from molecular profiles using machine learning is crucial for this goal. However, the high dimensionality of the molecular profiles compared to the limited number of samples presents significant challenges. Knowledge-based feature selection methods are particularly suitable for drug response prediction, as they leverage biological insights to reduce dimensionality and improve model interpretability. This study presents the first comparative evaluation of nine different knowledge-based and data-driven feature reduction methods on cell line and tumor data. Our analysis employs six distinct machine learning models, with a total of more than 6,000 runs to ensure a robust evaluation. Our findings indicate that transcription factor activities outperform other methods in predicting drug responses, effectively distinguishing between sensitive and resistant tumors for seven of the 20 drugs evaluated.

**Supplementary Information:**

The online version contains supplementary material available at 10.1038/s41598-024-81866-1.

## Introduction

Precision medicine has emerged as a promising approach to revolutionize healthcare by tailoring treatment to individual patients^1,2^. For certain pathologies, such as breast cancer, the discovery of key molecular differences between patients has allowed the development of personalized therapies that improve significantly over the previous one-size-fits-all approach. For other pathologies, such as ovarian cancer or many autoimmune disorders, significant patient heterogeneity is evident, but biomarkers that would indicate whether a given drug would be effective for a given patient are missing.

The rapid development of molecular profiling technologies has spurred interest in *machine learning* (ML) techniques that could be used to discover such biomarkers and their combinations in an automated fashion. Here, we refer to the problem of identifying these biomarkers from molecular profiling data as *drug response prediction* (DRP). Most of the available data on drug effects exist for cancer cell lines and tumor samples. The development of ML models for DRP in cancer is heavily based on publicly available drug screening databases that provide molecular profiles of cell lines, such as gene expression and their phenotypic responses to various drugs. By screening thousands of compounds across 59 cell lines, the *NCI-60 project*^3^ paved the way for efforts such as the *Genomics of Drug Sensitivity in Cancer* (GDSC)^4,5^ and the *Cancer Cell Line Encyclopedia* (CCLE)^6^, both of which are based on a larger number of cell lines but few drugs. More recently, the PRISM database has emerged as a comprehensive resource for drug screening, providing a rich dataset that covers a wide range of cancer and noncancer drugs, and an extensive collection of cancer cell lines^7^.

Based on these datasets, various ML models have been developed to predict drug responses in cell lines according to their molecular profiles^8–10^. Unlike many other ML applications, highly useful ML models for DRP need not only to make accurate predictions, but also to be easily interpretable, which is important for several reasons. First, predictions of interpretable models can be considered much more easily together with the often rich contextual knowledge, which, in turn, can give rise to a refined assessment of drug action or testable hypotheses. Both are extremely valuable given the high hurdles involved in the testing of drugs in humans. Second, the behavior and limitations of the model itself can also be evaluated much better in this rich context.

Some ML models with the best prediction performance are too complex to be easily interpreted. For example, the impact of a change in a single input feature on the output of a nonlinear deep neural network model tends to be extremely complex—certainly when compared to a simple linear model, where the impact fully depends transparently on a single model parameter. A second hurdle to interpretability is the high number of input features, which typically leads to highly complex interpretations in the context of biological knowledge^11,12^. Feature reduction (FR), i.e. the process of reducing the number of features, can thus improve model interpretability but also the predictive performance of the model, due to the removal of redundant information, especially when the sample size is limited.

Among the different types of molecular profiling data, gene expression has been found to be the most informative single data type for DRP^5,13–17^, and most DRP methods are based on this type of data. Several feature selection techniques have been applied to gene expression data in the context of DRP, but few studies have evaluated and compared these methods within a unified framework^18–20^. Parca et al.^18^ used two feature selection methods, *drug-unspecific genes* (DUG) and *drug-specific genes* (DSG). DUG is based on a set of genes that vary the most, while DSG is based on genes whose expression is highly correlated with drug response across training samples. Koras et al.^19^ proposed a framework to compare different knowledge-based feature selection methods (i.e., selection based on prior domain-specific knowledge) with data-driven feature selection methods (i.e., based on patterns in experimental data). Their data-driven features were obtained using *lasso* and *random forest* (RF), while their knowledge-based features included genes related to *drug target pathways* and known biological mechanisms. However, these comparative studies were limited to data from cell lines. Although cell lines provide rich information on drug mechanisms of action^3,21^, they may not be adequate for modeling critical aspects of cancer tumors, such as microenvironment interactions and heterogeneity^22,23^, which may limit the significance of cell line-based findings. Schätzle et al.^20^ compared the performance of ML models using three different feature sets, including highly variable genes, differentially expressed genes between the most sensitive and resistant tumors, and a predefined gene set derived from the *LINCS-L1000* project, known as *Landmark genes*^24^. All of the above studies were limited to methods that select a subset of genes and did not consider other methods to transform features into a low-dimensional space.

**Fig. 1:**
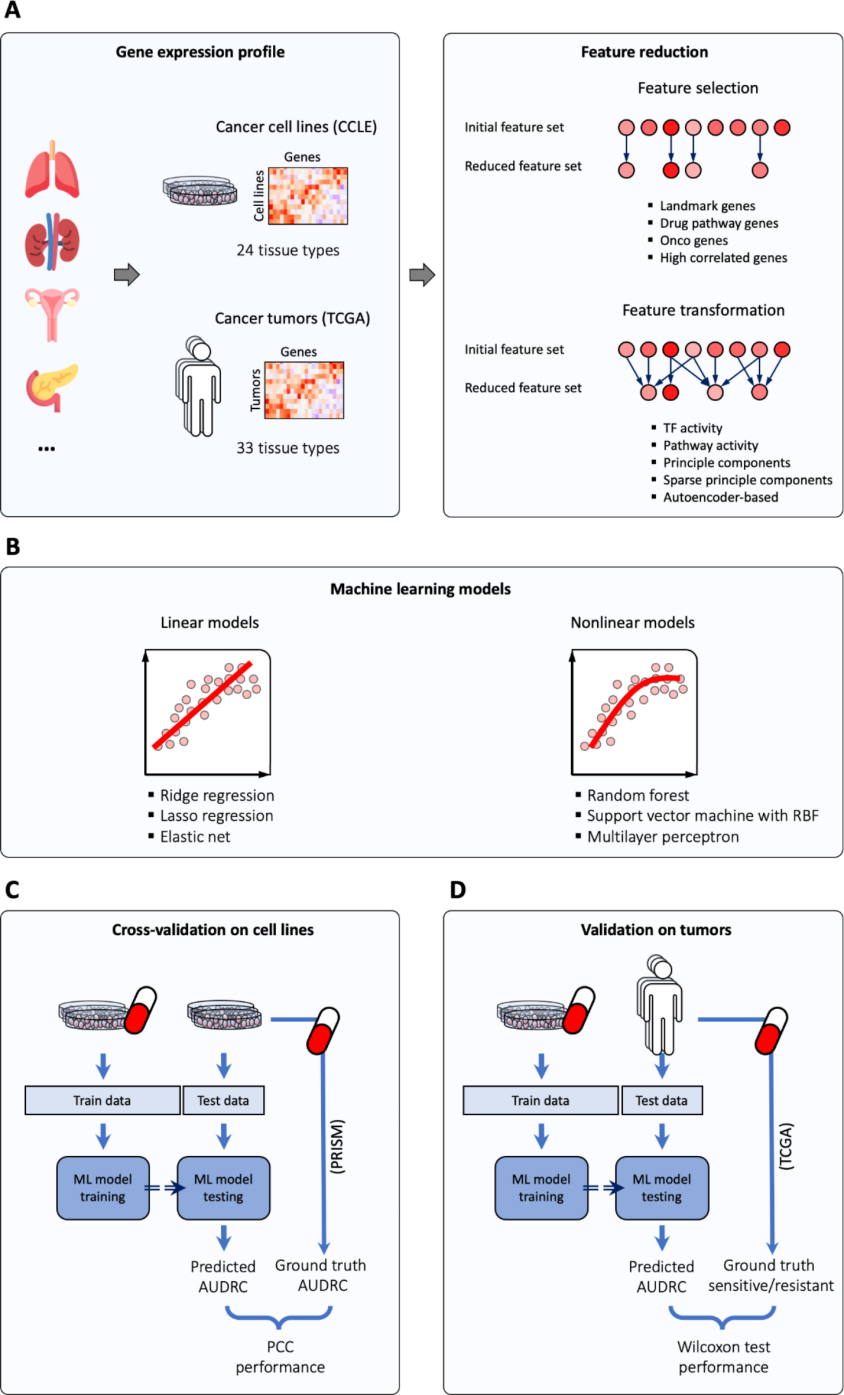
Our workflow to assess the performance of feature reduction methods. (**A**) The dimensionality of gene expression profiles of cancer cell lines and tumors is reduced using various feature reduction methods. (**B**) Each reduced feature set is then provided to a set of linear and nonlinear machine learning (ML) models for drug response prediction. To assess predictive performance for each combination of feature reduction method and ML model, two approaches were used: (**C**) cross-validation on cell line data, where the ML models are trained and tested on distinct sets of cell lines (PRISM dataset); and (**D**) validation where the ML models are trained on cell lines and tested on tumor data (TCGA dataset). AUDRC: area under the dose-response curve, PCC: Pearson correlation coefficient.

In this study, we evaluated the performance of nine different knowledge-based and data-driven FR methods for DRP on cancer cell line and tumor transcriptomes. FR can be data-driven or knowledge-based. In particular, we consider knowledge-based FR methods, which tend to provide better interpretability, and thus discoverability of the biological mechanisms underlying drug action. We selected five commonly used canonical knowledge-based FR methods and four canonical data-driven FR methods from two broader classes (Fig. 1A; Table 1):

- *Feature selection.* In feature selection, the most relevant features are selected for downstream ML modeling. We evaluated four commonly used feature selection strategies for DRP: *Landmark genes* from the *LINCS-L1000* project^24^: a set of genes that capture a significant amount of information in the entire transcriptome; *Drug pathway genes*, i.e., *All gene expressions* within known pathways (e.g., *Reactome* pathways^25^) that contain targets for a particular drug; OncoKB genes^26^: a curated resource of clinically actionable cancer genes; *highly correlated genes* (HCG): the set of genes that are highly correlated with the responses to specific drugs in the training set.
- *Feature transformation.* In this approach, features are projected into a low-dimensional space using a linear or nonlinear function, rather than being just selected. In this category, we include three data-driven and two knowledge-based feature transformations: *Top principal components (PCs)*,* a linear transformation that captures a maximum of variance in the data with a minimum number of dimensions; Top sparse PCs (SPCs)*, a linear transformation that preserves feature sparsity while reducing dimensionality; *Autoencoder embedding* (AE), a nonlinear transformation learned by autoencoders—a type of feed forward neural networks—to capture nonlinear data patterns in a reduced representation; *Pathway activities*^27^, a set of scores that quantify the activity of pathways based on the expressions of genes related to their downstream pathway; and *transcription factor* (TF*) activities*^28,29^, a set of scores that quantify the activity of TFs based on the expression of the genes they are known to regulate. Although *Pathway activities* have previously been used for DRP of cell lines^37^, their application to tumor data has not yet been studied. Moreover, to our knowledge, this is the first study to evaluate *TF activities* as a feature reduction method for DRP.

Once feature reduction has been applied, we evaluate the performance of feature reduction methods for single-drug learning by feeding their output to canonical versions of the most commonly used ML models in this context^8^: ridge regression, lasso regression, elastic net, support vector machine (SVM), multilayer perceptron (MLP) and RF (Fig. 1B). Note that we did not include more advanced deep learning (DL) models, as the datasets that would be available for a systematic comparison are not available today for models of this type, as in the case of the prediction of tumor responses we discuss in Sect. Cross-validation on cell lines. Our analysis workflow is illustrated in Fig. 1.

**Table 1:** Feature reduction methods used in our study.

	Knowledge-based	Data-Driven
Feature selection	• Landmark genes • Drug pathway genes • OncoKB genes	• Highly correlated genes
Feature transformation	• Pathway activities • Transcription factor activities	• Top principal components • Top sparse principal components • Autoencoder Embedding

## Results

**Fig. 2:**
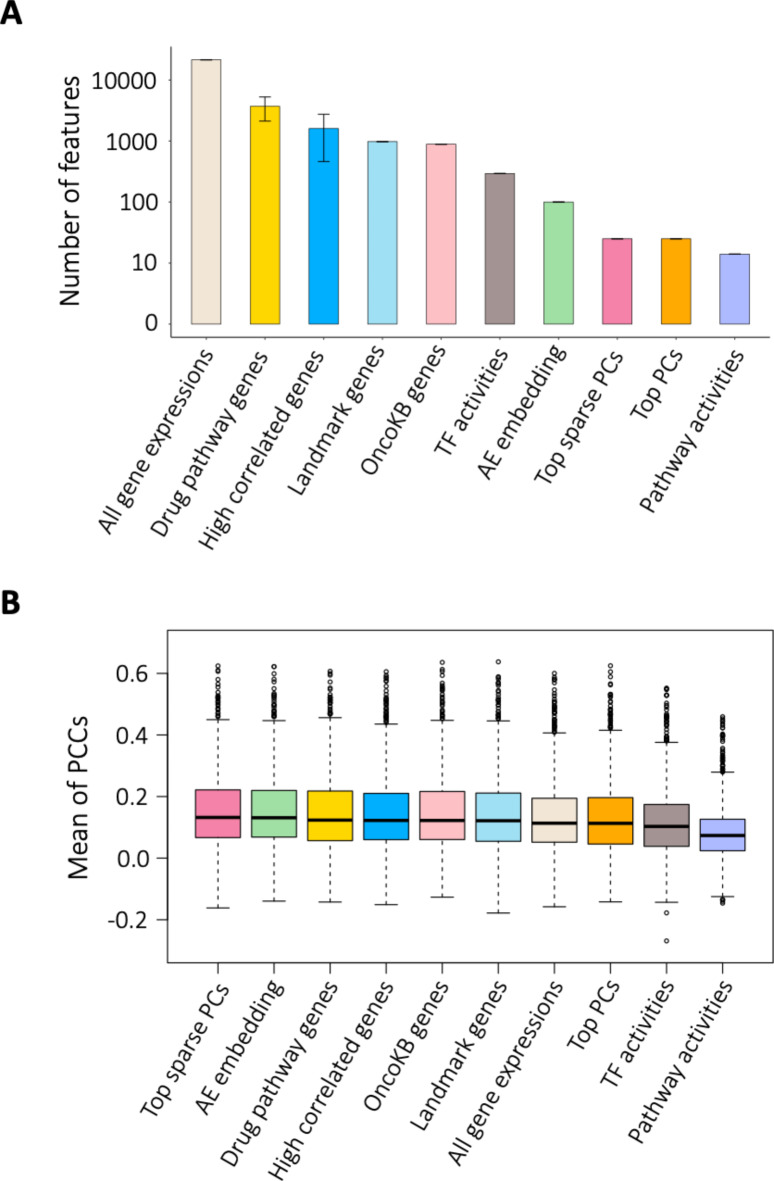
Comparison of different feature reduction methods through cross-validation on cell lines. (**A**) Number of features for each feature reduction method. (**B**) Cross-validation performance on cell lines using ridge regression in terms of Pearson’s correlation coefficient (PCC) on different feature reduction methods. TF stands for transcription factor.

Our base input data set consists of 21,408 gene expression measurements for each of the 1,094 cell lines provided in the CCLE data set^30^. After applying different feature reduction methods, we obtained different sets of features whose sizes varied significantly (Fig. 2A). *Drug pathway genes*, with an average of 3,704 genes (ranging from 148 to 7,625 features across different drugs), had the highest number of features, while *Pathway activities* had the fewest, with only 14 features. As a baseline, we also included *All gene expressions* that represents no feature reduction. The large spectrum of sizes of feature sets allows us to also analyze model performance with respect to feature count and potential redundancies that may exist among them.

We evaluated DRP performance in two different ways. First, we performed a cross-validation analysis on cell lines, where the training and test sets are random subsets of the cell line data (Fig. 1C). Second, we use the more ambitious and practically relevant validation on tumors, where the train and test sets are cell line data and clinical tumor data (Fig. 1D), respectively. Further details on the datasets are provided in Sect. 5: *Materials and Methods*.

### Cross-validation on cell lines

Our cross-validation analysis is based on the expression profiles of 480 cancer cell lines across 24 tissue types, as well as their responses to more than 1,400 drugs from the (relatively recent) PRISM dataset. Responses are provided as the *area under the dose-response curves* (AUC), a common measure of the overall drug sensitivity of a cell line to a particular drug. We note that many published studies focus on older drug response data for smaller sets of drugs (e.g., GDSC and CCLE datasets). In our study, we preferred the newer, broader and potentially less biased PRISM dataset, even if the nominal DRP performance may not be comparable to previous studies.

To robustly measure the predictive performance for any given combination of feature reduction and ML method, we implemented repeated random-subsampling cross-validation, as follows. We randomly split the data 100 times into 80% for training and 20% for testing, and reported the empirical model performance as the average Pearson’s correlation coefficient (PCC) between predicted and ground-truth drug responses. To determine any required hyperparameters, we used nested five-fold cross-validation in the training data (see Materials and Methods).

When comparing the performance of different ML models, we found that ridge regression performs at least as well as any other ML model, independently of the feature reduction method used (Figure S1). The other models, in order of decreasing performance, were RF, MLP, SVM, elastic net, and lasso. Figure 2B shows the performance of different feature reduction methods when followed by ridge regression. We did not observe significant differences between the empirical performances of *Top sparse PCs*,* AE embedding*,* Drug pathway genes*,* High correlated genes*,* OncoKB genes*, and *Landmark genes*. However, Top sparse PCs yielded significantly better performance than *All gene expressions*,* while* no significant difference was observed between *Landmark genes and All gene expressions. All gene expressions* and *Top PCs performed* similarly to each other, while outperforming *TF activities* and *Pathway activities.* Lastly, *Pathway activities* was significantly worse than all the other methods. All tests were performed using the Wilcoxon rank sum test with a significance level of 0.05.

We note that, compared to other studies, the overall DRP performance appears to be relatively low. The fact that our evaluation incorporates all drugs from the large PRISM dataset (as discussed above) may be a contributing factor. Additionally, our focus on combinations of canonical features and ML methods may leave room for improved DRP performance here and there. Yet, we believe that, given the currently still limited amounts of data, a comparison of a few canonical methods, such as the one we present here, yields the most meaningful and robust conclusions about the comparative performance of canonical feature reduction methods that the field needs at this point.

### Validation on tumor samples

Until now, by far most drug response data are available from cell lines, and most DRP ML studies are based on these data. Tumor samples represent more of the clinical complexity of cancer, but much less molecular profiling and drug response data are available. To evaluate the performance of DRP in a more clinically relevant setting, we trained models on all available cell line data using the same feature reduction and ML methods as above, but evaluated their performance on transcriptome and drug response data from tumor samples.

Specifically, we trained ML models on all data from PRISM cell lines for each drug and subsequently tested their performance on *TCGA* human tumor data (transcriptomes and drug responses) that come from 33 different types of cancer^21^. TCGA drug responses are classified into four classes: *complete response*, *partial response*, *progressive disease*, and *stable disease*. To allow a conceptually simple comparison between the feature reduction methods in this scenario, we reduced the four classes to two by labeling the first two classes as *sensitive* and the last two as *resistant*. There are 59 drugs common to the TCGA and PRISM data sets, among which 20 drugs have measured drug responses for at least 5 samples in each of the sensitive and resistant classes. We note that the drug responses of cell line-trained models are predicted as a score, whereas the tumor response is binary. To assess whether the prediction model can distinguish between sensitive and resistant tumors, we tested whether the predicted scores were significantly lower for sensitive tumors than for resistant tumors using a one-sided *Mann-Whitney-Wilcoxon* test with a minimum significance level of 0.05.

**Fig. 3:**
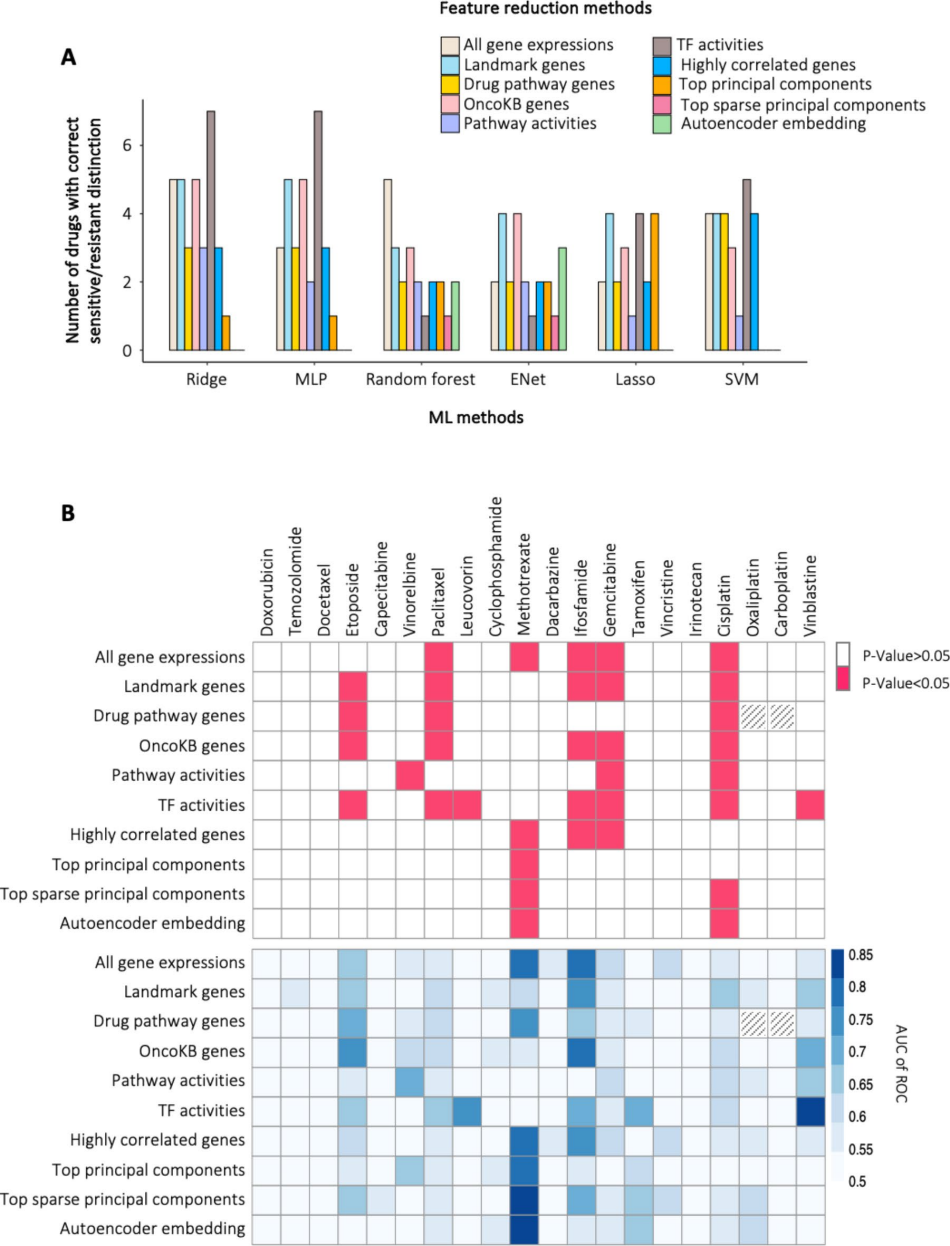
The ability of different feature reduction methods to distinguish sensitive from resistant tumors. (**A**) The number of drugs, out of 20, for which sensitive and resistant tumors can be successfully distinguished using specific combinations of feature reduction method and ML method. ML methods: ridge regression, multilayer perceptron (MLP), random forest, elastic net (ENet), lasso, and support vector machine (SVM), (**B**) Drug-specific results for ridge regression, as the overall best ML model. [Top] *P-*values for the one-sided *Mann-Whitney-Wilcoxon* test and [Bottom] area under the *ROC* curve when classifying tumors using predicted drug responses. Hash patterns indicate cases where no drug targets were available in *Drug pathway genes* feature reduction method. TF stands for the transcription factor.

Fig. 3A shows, for each ML method, the number of drugs for which each feature reduction method was able to distinguish (in the statistical sense) sensitive from resistant tumors. Interestingly, and similar to the case of cell lines, ridge regression outperforms the other ML models and consistently achieves the best predictive performance across all feature reduction methods. This observation is consistent with the idea that ridge regression is particularly suited for scenarios with highly correlated input variables corresponding, in our case, to the well-known phenomenon of sets of coexpressed genes.

Among the feature reduction methods, we observed that *TF activities* provided the best predictive performance on tumor data (seven out of 20 drugs). *Landmark* genes and *OncoKB* genes were ranked as the second-best methods, correctly distinguishing responses to five drugs and performing equally to using *All gene expressions.* It is worth mentioning that *TF activities* are a significantly smaller feature set than *OncoKB* genes, *Landmark* genes, and *All gene expressions* (293 genes vs. 887, 978 and 21,408 genes, respectively). The performance of the feature reduction methods for each drug is shown in Fig. 3B and Table S1, when used with ridge regression, and in Figure S3 and Table S2-6, when used with the other ML models.

Interestingly, the most successful feature reduction methods are those that focus on specific genes that may play an *a priori* role in controlling drug effects. Indeed, many genes are known to be drug targets, they regulate drug metabolism or transport, or they have specific roles in specific cell types, such as cancer cells, including modulation of signaling pathways relevant to growth, apoptosis, and differentiation. We note that a significant number of drugs are not classifiable using any of the FR methods tested and that the FR methods that correctly classify the effects of methotrexate are quite distinct from the other drugs we tested. This suggests that the best FR method may depend on the drug with which it is to be used.

To assess whether highly performing models might also have a biological interpretation, we further analyzed the globally highly performing combination of *TF activities* and ridge regression. For those seven drugs for which ridge regression with *TF activities* correctly distinguished sensitive from resistant tumors the ten top TFs with the largest absolute model coefficients are listed in (Table S7). Interestingly, several of these TFs have already been reported as important regulators in the literature. For example, the study by Sakai et al.^31^ suggested that high expression of IRF1 is associated with high sensitivity of pancreatic cancer cells to gemcitabine. More recently, overexpression of HOXB13 and NFE2L1 has been reported to be associated with tumor resistance to cisplatin^32–34^.

## Discussion

In most computational analyses of high-dimensional molecular profiling datasets, such as transcriptomic data, feature reduction is a crucial first step that strongly conditions prediction performance and interpretability. The present study represents the first systematic comparison of data-driven and knowledge-based feature reduction methods for their ability to predict drug responses on cell lines and tumors. Our evaluation included cross-validation across more than 1,400 drugs in the recently published PRISM cell line dataset^7^, as well as an evaluation of PRISM-trained models on 33 different tumors from TCGA.

When combined with six ML models, we found that *top sparse principal components*, *Drug pathway genes*, and *Landmark genes* performed better than other feature reduction methods for predicting drug responses in cell lines. In the biologically much more complex case of clinical tumors, *TF activities* performed the best. In both scenarios, ridge regression was the best machine learning model across the different feature reduction methods, suggesting its strength in handling the correlated features typical in gene expression data. Our detailed analysis of the combination of *TF activities* and ridge regression led to a model based on several transcription factors, such as IRF1^35^ and NFE2L1^36–38^, which have known roles in drug response, as important predictors in the DRP model for specific drugs. These findings highlight the potential of *TF activities* as interpretable and biologically relevant predictors in DRP.

The differences we observed in the performance between feature reduction methods between cell lines and tumor samples may have many biological causes. Importantly, tumor tissues exhibit complex interactions within the microenvironment and cellular heterogeneity that influence drug response, whereas cell lines are simplified systems without this level of complexity. It is possible that the better performance of *TF activities* stems from its robustness to the likely higher biological and technical variability in tumors and its ability to capture the state of the more complex regulatory networks in tumors.

It is important to note that, in addition to biological differences, all of the current handful of drug response datasets are also biased through inconsistencies in sample preparation, data processing, and drug response measurement, and the impact of these biases on our results is equally difficult to assess. Therefore, not only future larger datasets, but also higher degrees of standardization will be key to increase the robustness of insights about feature reduction methods for drug response prediction for cell lines, tumors, and perhaps also organoid models of intermediate biological complexity.

The superiority of the *TF activities* feature reduction method in our empirical study on tumors raises other questions that can be addressed in further research. Assuming that our results can be confirmed in independent datasets, can the field of transcription factors be further narrowed, perhaps using other types of experimental data of knowledge, in drug-dependent ways? Can the activity profile of transcription factors be interpreted as a cellular state that could also be useful for other prediction tasks? Might it be possible to refine the characterization of *TF activities based on* pertinent complementary profiling data, such as post-translational modifications? If so, can experiments be simplified and targeted to obtain this information? Finally, after we observed (in Fig. 3B) that a suitable FR method may depend on the drug with which it is to be used, it may also be interesting to better understand this dependency. The corresponding insights may then be used in ML methods that use drug features as additional input^39,40^.

When placed in the specific context of DRP, some generic feature reduction approaches we studied offer potential for future improvement. To give an example, in the evaluation of the *Drug pathway genes* method, we selected genes within the generic ‘root’ Reactome pathways that contain the targets for each drug. However, other choices are possible, as Reactome has a hierarchical pathway structure such that each drug target may exist in multiple nested pathways that capture the biology underlying DRP at multiple levels of biological resolution. Finer levels of representation than the ‘root’ pathways may well lead to better DRP performance, by better reducing feature redundancy and improving interpretability. The systematic methodological improvement of the *Drug pathway genes* feature reduction method through data- or also knowledge-based approaches is only one of the ways in which feature selection methods for drug response prediction stand to evolve in the future.

Feature reduction methods are also expected to evolve along with experimental technologies that capture the biology underlying DRP with greater precision. *TF activities*, for example, are based on the expression of the target genes that they are believed to regulate. However, the target genes of TFs are often known only approximately and, furthermore, the idea that a given TF always regulates the same set of target genes is a strong simplification. Tumor tissue, for example, is often a mixture of cell types with potentially very different sets of TF target genes regulated by the same TF. Single cell profiling technologies may be needed to understand, and treat, cell type- and patient-specific resistance mechanisms. Furthermore, even in cells of the same cell type, variations in the nutrient content and the concentration of signaling molecules in the cellular environment can modify the dynamics of TF through variations in the state of chromatin, the expression of cofactors and other interacting proteins, post-translational modifications, and differential expression of TF isoforms, to name a few possibilities. We expect that the performance of *TF activities*-based drug response prediction will improve together with the development of molecular profiling technologies that can provide ML-based approaches with specific new relevant biological features that lead to better predictions and explanations of drug responses.

Finally, higher-level states of molecular network *motifs*, e.g^41,42^, especially when projected into a lower-dimensional space, may constitute features that capture molecular variation associated with variation in drug response better than single gene expression values. We expect that the close interaction between the accessibility of new molecular profiling technologies, new large datasets, the development of new computational methods, and the discovery of new network motifs will work hand in hand with feature reduction methods to advance drug response prediction toward routine clinical applications.

## Materials and methods

### Data sets

**Data from cell lines.** The PRISM dataset^7^ covers the responses of 4,518 drugs in 480 human cancer cell lines from the CCLE project^6^. RNA-seq gene expression profiles were downloaded from https://sites.broadinstitute.org/ccle. Drug sensitivities of the secondary screen, including the parameters of the dose-response curve of 1,448 drugs, were downloaded from https://depmap.org/repurposing/.

**Data from tumor samples.** The Cancer Genome Atlas (TCGA)^21^ is a collaborative effort between the National Cancer Institute and the National Human Genome Research Institute. TCGA provides molecular profiles, including mRNA, miRNA, reverse phase protein arrays, DNA methylation, copy number alterations, and gene mutation, of primary tumors for thousands of patients with 33 types of cancer. Here, we used the mRNA-seq profiles of TCGA tumors, which are freely accessible through the FireBrowse website (http://firebrowse.org). For some tumors, the response to treatment is available and classified into *complete response*, *partial response*, *progressive disease*, and *stable disease* according to the RECIST imaging-based measure of tumor growth (i.e., an imaging-based criterion to evaluate the response in solid tumors^43^. Tumor response data were the ones provided in the Supplementary Material of Ding et al.^44^.

### Pre-processing

For both the cell line and the tumor transcriptome profile, we downloaded the RSEM-normalized gene expression data (TPM) and then performed a log2-transformation after adding 1 to each count value$\:{\:log}_{2}(TPM+1)$. Then, for normalization between samples, we used a gene-wise z score transformation, that is, z-score normalization of gene expressions for each gene across all samples. Furthermore, to test ML models on tumors, we used *Combat* (*R* package *sva version 3.46*)^45^ to remove any batch effect between cell lines and tumor data.

## Feature reduction methods

### Feature selection

***Landmark genes***. Subramanian et al.^24^ showed that a set of 978 *Landmark* genes is largely sufficient to capture the variation of the entire transcriptome, and up to 80% of the expected regulatory network connections between more than 12,000 genes can be correctly inferred. The *Landmark* genes are publicly available on both the Gene Expression Omnibus repository (GEO: GSE92742) and the *CMap* linked user environment (CLUE) (https://clue.io/releases/data-dashboard*).*

***Drug pathway genes***. For many drugs, prior information about their target proteins exists, each of which interacts with other molecules through recognized pathways. For each given drug, we obtained a list of targets from the PRISM dataset, selected the Reactome ‘root’ pathways containing one or more target genes and used *All gene expressions* within these pathways as features (Reactome version 82 released in September 2022).

**OncoKB genes.** a curated resource of clinically actionable cancer genes, based on their inclusion in various sequencing panels, the Sanger Cancer Gene Census, etc. *OncoKB genes* contains 1066 genes that are publicly available at https://www.oncokb.org/.

**Highly correlated genes.** We selected genes whose expressions are highly correlated with the drug response. To this end, for each drug, we performed a correlation test (using cor.test() function in R) between the drug response and the expressions of *All gene expressions* on the training set. The genes with the p-value < 0.05 were selected as features.

### Feature transformation

***Pathway activities***. We calculated *Pathway activities* using the *PROGENy* R package (*version 1.20)*^27^, a method to infer the signaling activity of pathways from gene expression data using downstream signatures. PROGENy computes activities for 14 cancer-related pathways, including *Androgen*, *EGFR*, *Estrogen*, *Hypoxia*, *JAK-STAT*, *MAPK*, *NFkB*, *p53*, *PI3K*, *TGFb*, *TNFa*, *Trail*, *VEGF*, and *WNT*.

***TF activities***. TFs are a relatively small class of proteins that regulate the expression of potentially many other genes, and their dysregulation can play a key role in tumor progression, metastasis, and drug resistance^46,47^. Therefore, a set of *TF activities* can be interpreted as a compact representation of the functional cellular state. A set of *TF activities* can be obtained using a *footprint*-based method^48^, where *TF activities* are inferred from the expression of the genes they target. Garcia et al.^29^, derived a collection of consensus TF-target interactions based on different sources, including TF binding site predictions, curated annotations, annotations from automatic text mining, and chromatin immunoprecipitation, and constructed a network of identified TF-target interactions called *DoRothEA*^29^. In the present study, we used *decoupleR*^28^ *version 2.4* with the GSEA option for the calculation of *TF activities.*

**Principal components.** To obtain PCs, we applied a PCA on the expressions of *All gene expressions* (using prcomp() function in R). The number of PCs was set to 50, corresponding to the elbow point on the explained variance curve.

**Sparse Principal components.** The PCA method was extended to SPCA implemented with the *sparsepca* R package (*v0.1*)^49^. Parameters were fine-tuned on a validation set to achieve an optimal balance between sparsity and explained variance. Specifically, the key hyperparameters, alpha and beta, which control the sparsity of coefficients and the number of nonzero coefficients, respectively, were selected based on the best observed performance on validation data. The number of components was set to 25, corresponding to the elbow point on the explained variance curve.

**Autoencoder embedding.** We implemented a shallow autoencoder based on the *keras* R package (*v2.15*). The number of embedding dimensions was set to 100 because it resulted in the smallest mean squared error in reconstructing gene expressions. The autoencoder model was trained using the Adam optimizer and the mean squared error loss function over 20 epochs with a batch size of 32 and a validation split of 20%.

### Machine learning models

We implemented three linear ML models (ridge regression, lasso regression, and elastic net) and three nonlinear ones (i.e., RF, SVM, and MLP). To learn more about the specifics of these ML models, we refer the reader to Hastie et al.^50,51^. For linear models, we used the implementations of the R package *caret (version 6.0)*^52^. The linear models we used incorporate regularization terms in their cost functions (L1 for Lasso, L2 for Ridge, and both L1 and L2 for ElasticNet) that introduce bias towards sparse solutions and thus improve the suitability for datasets with a high number of features. To optimize any hyperparameters, we employed nested cross-validation, split the training data into validation and training sets, and selected hyperparameters for optimal performance on the validation set. Independently for each drug, we performed a five-fold nested cross-validation approach to obtain the optimum regularization parameters. Our implementation of RF is based on the *randomForest*^53^ R package *(version 4.7)* with 150 trees and 100 randomly sampled candidate features at each split. We observed that the RF outcomes were not too sensitive to variation in the above values of these hyperparameters. We used SVM implementation of the *caret* package (version *6.0*). Although the most basic version of SVM uses a linear model, it can accommodate nonlinearity through kernel functions. We selected a nonlinear SVM with the most commonly used *radial basis function* (*RBF*) kernel, because we sought to diversify our model set beyond linear models while adhering to time constraints. The hyperparameters we tuned for the SVM model were the box constraint (as a regularization parameter) and kernel width. To tune these hyperparameters, we used the same nested cross-validation framework as for the linear models. Finally, we used the MLP implementation of the R package *keras (version 2.11)*, with two hidden layers of 150 and 100 neurons, respectively. The activation functions of the hidden layers and the output layer were *Sigmoid* and *Linear*, respectively. As we observed that the performance was not sensitive to variations in the number of hidden neurons, we did not include their number as a tuneable hyperparameter.

### Key Points

- Feature reduction methods are critical for machine learning of models that predict drug responses from high-dimensional molecular profiling data.
- The present article systematically evaluates the performance of nine feature reduction methods in the context of machine learning methods for drug response prediction.
- In the clinically relevant case of tumor molecular profile data, *Transcription Factor Activities* performed best, suggesting that a few hundred estimated transcription factor activities contain key information related to drug response.
- The source code provided can be used to reproduce and extend the results of this article.

## Electronic supplementary material

Below is the link to the electronic supplementary material.

Supplementary Material 1

## Data Availability

Cell line gene expression data and drug sensitivities are publicly available at https://sites.broadinstitute.org/ccle and https://depmap.org/repurposing/, respectively. The tumor mRNA sequencing profiles of TCGA are accessible through the FireBrowse website http://firebrowse.org, and their drug are the Supplementary Data provided by Ding et al. [36]. The open source codes that allow the reader to recapitulate and extend the analysis are available through the GitHub repository https://github.com/faren-f/FS4DRP/tree/master.
