# Supplementary material for: Comparative evaluation of feature reduction methods for drug response prediction
Source: Sci Rep. 2024 Dec 28;14:30885. doi: 10.1038/s41598-024-81866-1 (PMC11680677; doi:10.1038/s41598-024-81866-1)
Supplement: Supplementary file 1 — Supplementary Material 1 [file 41598_2024_81866_MOESM1_ESM.pdf]

Supplementary Material

Comparative evaluation of feature reduction methods for drug response prediction

A

Landmark genes	Ridge	MLP	RF	ENet	Lasso	SVM
Ridge			*** >	*** >	*** >	*** >
MLP			** >	*** >	*** >	*** >
RF				*** >	*** >	* >
ENet					*** >	
Lasso						
SVM				*** >	*** >	

B

Drug pathway genes	Ridge	MLP	RF	ENet	Lasso	SVM
Ridge				*** >	*** >	*** >
MLP				*** >	*** >	*** >
RF				*** >	*** >	*** >
ENet					*** >	
Lasso						
SVM					*** >	

C

Pathway activities	Ridge	MLP	RF	ENet	Lasso	SVM
Ridge		* >				
MLP						
RF						
ENet						
Lasso						
SVM						

D

TF activities	Ridge	MLP	RF	ENet	Lasso	SVM
Ridge		*** >	*** >	*** >	*** >	*** >
MLP				*** >	*** >	*** >
RF				*** >	*** >	*** >
ENet						
Lasso				* >		
SVM				*** >	*** >	

E

All gene expressions	Ridge	MLP	RF	ENet	Lasso	SVM
Ridge		*** >		*** >	*** >	
MLP					*** >	
RF		*** >		*** >	*** >	
ENet					*** >	
Lasso						
SVM		*** >		*** >	*** >	

F

OncoKB genes	Ridge	MLP	RF	ENet	Lasso	SVM
Ridge		*** >	*** >	*** >	*** >	*** >
MLP				*** >	*** >	** >
RF				*** >	*** >	* >
ENet					*** >	
Lasso						
SVM				*** >	*** >	

G

PCs	Ridge	MLP	RF	ENet	Lasso	SVM
Ridge		** >	* >			*** >
MLP			** >			
RF						* >
ENet		* >				*** >
Lasso		* >				** >
SVM						

H

SPCs	Ridge	MLP	RF	ENet	Lasso	SVM
Ridge		*** >	*** >	*** >	*** >	* >
MLP				*** >	*** >	
RF		* >		*** >	*** >	
ENet						
Lasso						
SVM		*** >	* >	*** >	*** >	

I

Autoencoder embedding	Ridge	MLP	RF	ENet	Lasso	SVM
Ridge		** >	*** >	*** >	*** >	*** >
MLP				*** >	*** >	*** >
RF				*** >	*** >	*** >
ENet						
Lasso						
SVM						

J

Highly correlated	Ridge	MLP	RF	ENet	Lasso	SVM
Ridge				*** >	*** >	* >
MLP				*** >	*** >	*** >
RF				*** >	*** >	** >
ENet					*** >	
Lasso						
SVM				*** >	*** >	

Figure S1. Pairwise performance comparisons among six different machine learning models, for input from different feature reduction methods: (A) *Landmark genes*, (B) *Drug pathway genes*, (C) *Pathway activities*, (D) *transcription factor (TF) activities*; and (E) *All gene expressions*. The greater (>) symbols indicate when the performance of the model in the corresponding row was found to be significantly better than the model in the corresponding column. Asterisks (*/**/***) indicate significance using the Mann-Whitney-Wilcoxon test (0.05/0.01/0.001). MLP: multilayer perceptron, RF: random forest, ENet: elastic net.

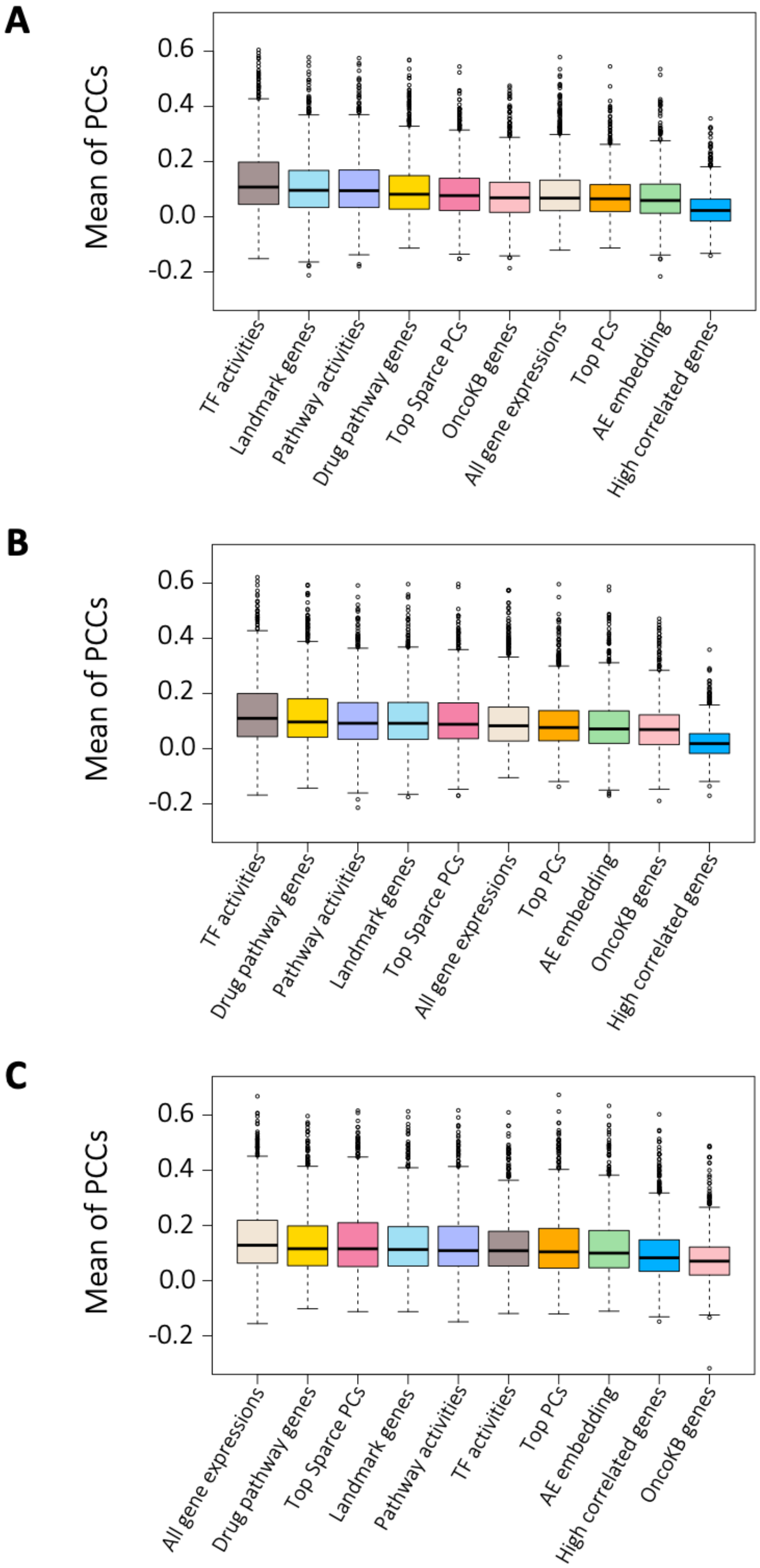
A
B
C
Mean of PCCs
0.6
0.4
0.2
0.0
-0.2
TF activities
Landmark genes
Pathway activities
Drug pathway genes
Top Sparce PCs
OncoKB genes
All gene expressions
Top PCs
AE embedding
High correlated genes

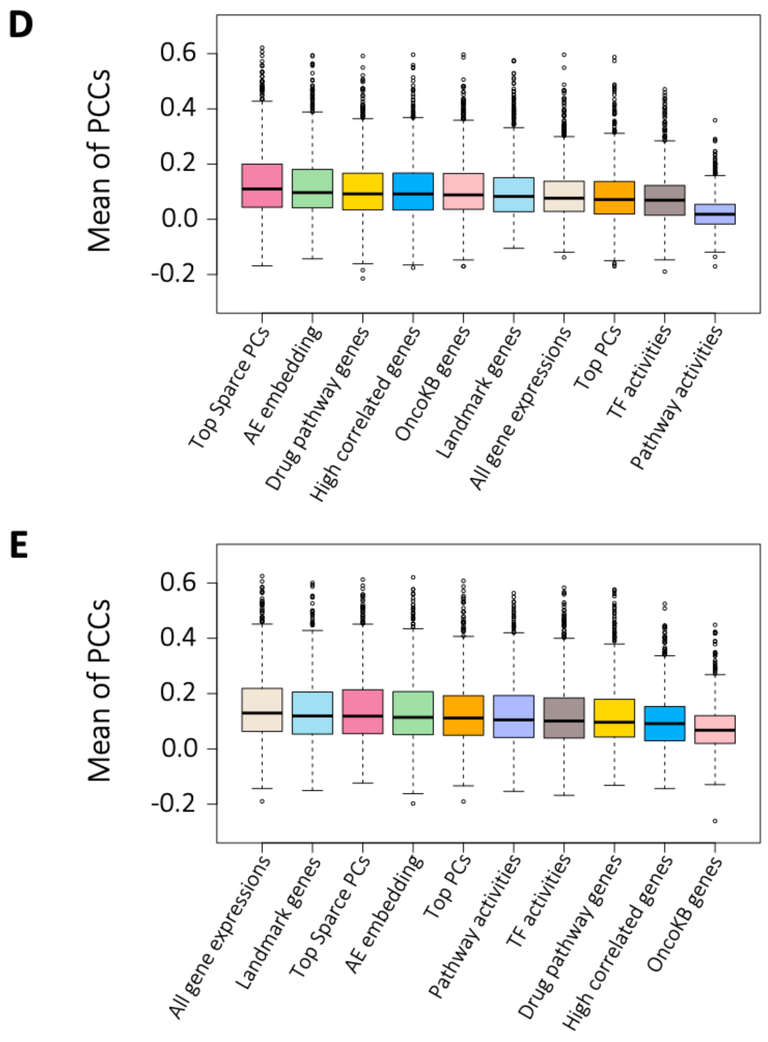

Figure S2. Performance of within-dataset analysis: Pearson correlation coefficient (PCC) over different feature reduction methods for different machine learning models: (A) lasso, (B) elastic net, (C) random forest, (D) support vector machine with radial basis function kernel, and (E) multilayer perceptron. Asterisks (*/**/***) indicate significance using the Mann-Whitney-Wilcoxon test (0.05/0.01/0.001).

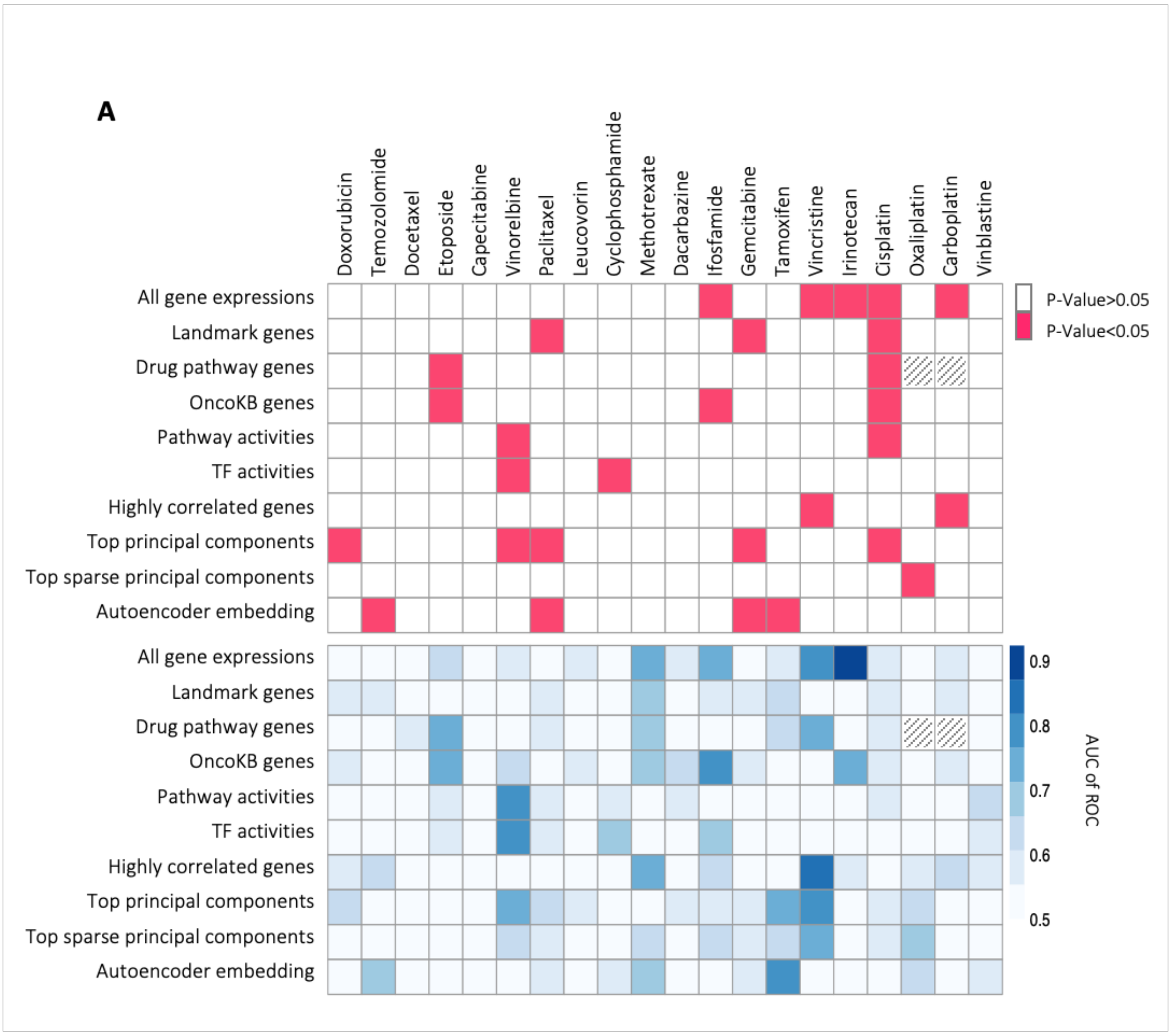

A
Doxorubicin
Temozolomide
Docetaxel
Etoposide
Capecitabine
Vinorelbine
Paclitaxel
Leucovorin
Cyclophosphamide
Methotrexate
Dacarbazine
Ifosfamide
Gemcitabine
Tamoxifen
Vincristine
Irinotecan
Cisplatin
Oxaliplatin
Carboplatin
Vinblastine
All gene expressions
Landmark genes
Drug pathway genes
OncoKB genes
Pathway activities
TF activities
Highly correlated genes
Top principal components
Top sparse principal components
Autoencoder embedding
P-Value>0.05
P-Value<0.05
0.9
0.8
0.7
0.6
0.5
AUC of ROC

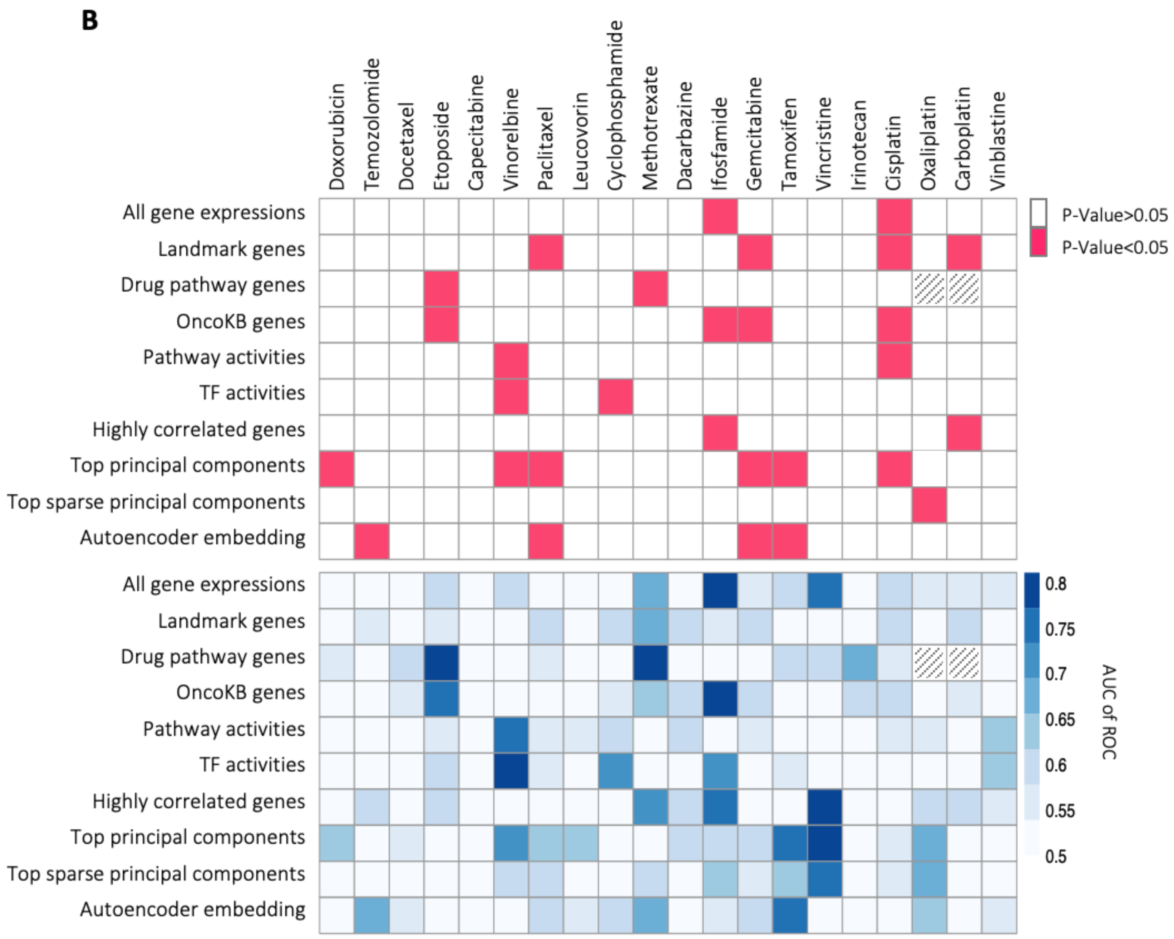

B
Doxorubicin
Temozolomide
Docetaxel
Etoposide
Capecitabine
Vinorelbine
Paclitaxel
Leucovorin
Cyclophosphamide
Methotrexate
Dacarbazine
Ifosfamide
Gemcitabine
Tamoxifen
Vincristine
Irinotecan
Cisplatin
Oxaliplatin
Carboplatin
Vinblastine
All gene expressions
Landmark genes
Drug pathway genes
OncoKB genes
Pathway activities
TF activities
Highly correlated genes
Top principal components
Top sparse principal components
Autoencoder embedding
P-Value>0.05
P-Value<0.05
0.8
0.75
0.7
0.65
0.6
0.55
0.5
AUC of ROC

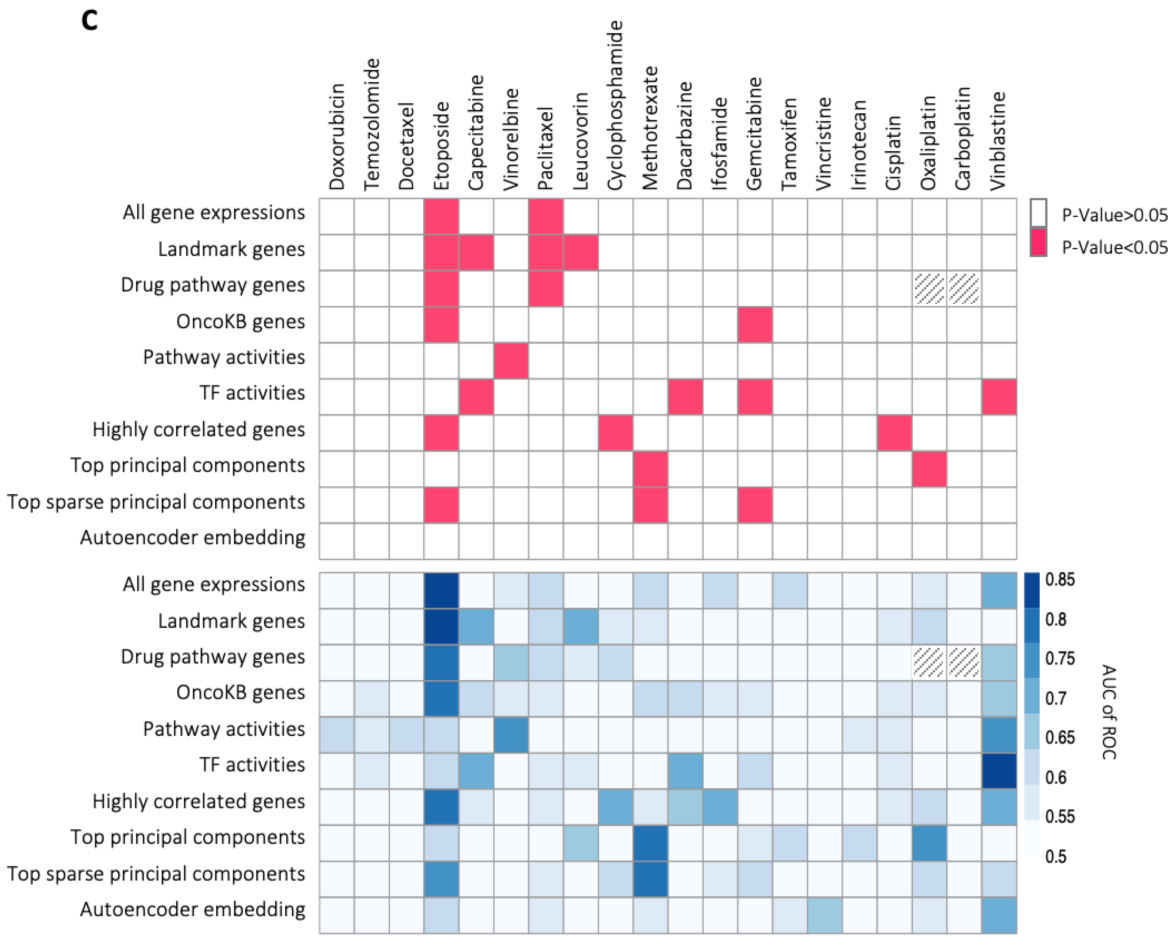

c
Doxorubicin
Temozolomide
Docetaxel
Etoposide
Capecitabine
Vinorelbine
Paclitaxel
Leucovorin
Cyclophosphamide
Methotrexate
Dacarbazine
Ifosfamide
Gemcitabine
Tamoxifen
Vincristine
Irinotecan
Cisplatin
Oxaliplatin
Carboplatin
Vinblastine
All gene expressions
Landmark genes
Drug pathway genes
OncoKB genes
Pathway activities
TF activities
Highly correlated genes
Top principal components
Top sparse principal components
Autoencoder embedding
P-Value>0.05
P-Value<0.05
0.85
0.8
0.75
0.7
0.65
0.6
0.55
0.5
AUC of ROC

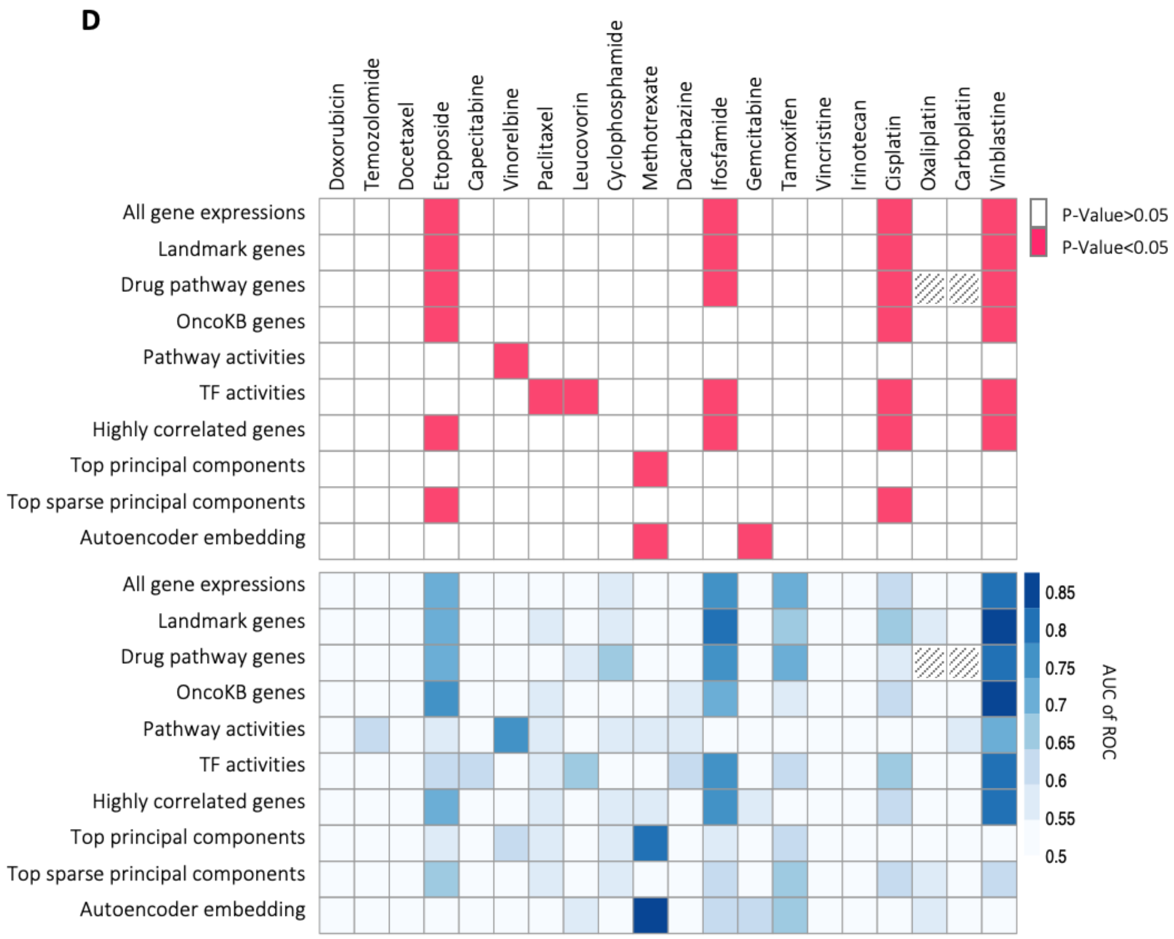
D
Doxorubicin
Temozolomide
Docetaxel
Etoposide
Capecitabine
Vinorelbine
Paclitaxel
Leucovorin
Cyclophosphamide
Methotrexate
Dacarbazine
Ifosfamide
Gemcitabine
Tamoxifen
Vincristine
Irinotecan
Cisplatin
Oxaliplatin
Carboplatin
Vinblastine
All gene expressions
Landmark genes
Drug pathway genes
OncoKB genes
Pathway activities
TF activities
Highly correlated genes
Top principal components
Top sparse principal components
Autoencoder embedding
P-Value>0.05
P-Value<0.05
0.85
0.8
0.75
0.7
0.65
0.6
0.55
0.5
AUC of ROC

E

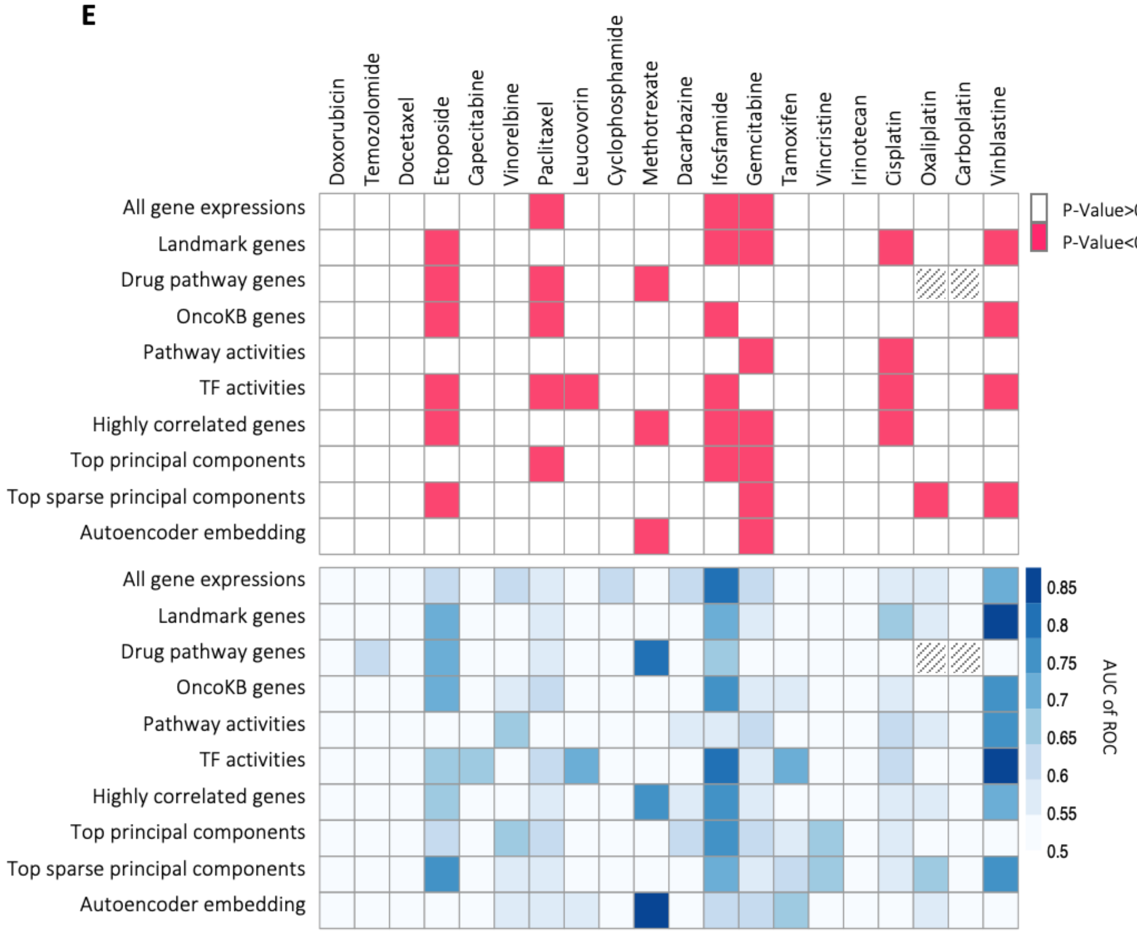

Figure S3. The results of cross-dataset analysis over different feature reduction methods in terms of [i] p-values of the one-sided Mann-Whitney-Wilcoxon test and [ii] area under the ROC curve for (A) lasso, (B) elastic net, (C) random forest, (D) support vector machine with radial basis function kernel, and (E) multilayer perceptron. The results for the shaded boxes have not been calculated due to the unavailability of the drug targets.

Table S1. The results of cross-dataset analysis over different feature reduction methods using ridge regression. Shown are p-values of a one-sided Mann-Whitney-Wilcoxon test for association between DRP prediction and ground truth.

	All gene expressions	Landmark genes	Drug pathway genes	OncoKB genes	Pathway activities	TF activities	Highly correlated genes	Top PCs	Top SPCs	Autoencoder embedding
**Doxorubicin**	0.77	0.93	0.85	0.97	0.84	0.76	0.57	0.58	0.98	0.96
**Temozolomide**	0.77	0.21	0.43	0.88	0.39	0.75	0.93	0.86	0.80	0.61
**Docetaxel**	0.26	0.75	0.57	0.45	0.48	0.33	0.38	0.36	0.97	0.68
**Etoposide**	0.08	0.03	0.01	0.00	0.25	0.02	0.11	0.27	0.07	0.48
**Capecitabine**	0.80	0.76	0.48	0.61	0.38	0.59	0.68	0.41	0.32	0.59
**Vinorelbine**	0.36	0.40	0.31	0.24	0.04	0.76	0.47	0.15	0.51	0.56
**Paclitaxel**	0.03	0.02	0.03	0.03	0.16	0.00	0.07	0.08	0.06	0.14
**Leucovorin**	0.76	0.55	0.52	0.73	0.50	0.00	0.67	0.48	0.64	0.36
**Cyclophosphamide**	0.54	0.31	0.68	0.34	0.58	0.43	0.65	0.24	0.53	0.28
**Methotrexate**	0.03	0.27	0.06	0.35	0.99	0.52	0.03	0.04	0.01	0.01
**Dacarbazine**	0.34	0.42	0.73	0.50	0.52	0.80	0.25	0.39	0.82	0.96
**Ifosfamide**	0.01	0.03	0.09	0.01	0.54	0.03	0.02	0.28	0.06	0.37
**Gemcitabine**	0.01	0.03	0.05	0.03	0.01	0.03	0.02	0.36	0.08	0.08
**Tamoxifen**	0.41	0.39	0.34	0.61	1.00	0.08	0.70	0.23	0.13	0.10
**Vincristine**	0.31	0.97	0.73	0.88	0.96	0.91	0.27	0.85	0.31	1.00
**Irinotecan**	0.76	0.99	0.98	0.76	0.85	0.98	0.66	0.71	0.96	0.91
**Cisplatin**	0.01	0.00	0.02	0.00	0.00	0.01	0.10	0.17	0.02	0.04
**Oxaliplatin**	0.47	0.19	–	0.69	0.30	0.78	0.21	0.40	0.11	0.17
**Carboplatin**	0.20	0.67	–	0.79	0.90	0.87	0.34	0.62	0.67	0.96
**Vinblastine**	0.30	0.15	0.30	0.13	0.15	0.01	0.38	0.80	0.50	0.58

Table S2. The results of cross-dataset analysis over different feature reduction methods using lasso regression. Shown are p-values of a one-sided Mann-Whitney-Wilcoxon test for association between DRP prediction and ground truth.

	All gene expressions	Landmark genes	Drug pathway genes	OncoKB genes	Pathway activities	TF activities	Highly correlated genes	Top PCs	Top SPCs	Autoencoder embedding
**Doxorubicin**	0.41	0.15	0.26	0.14	0.61	0.57	0.15	0.02	0.95	0.28
**Temozolomide**	0.98	0.29	0.82	0.91	0.49	0.82	0.09	0.70	0.79	0.03
**Docetaxel**	0.65	0.75	0.17	0.32	0.61	0.67	0.40	0.26	0.97	0.23
**Etoposide**	0.10	0.33	0.01	0.00	0.22	0.19	0.37	0.56	0.48	0.93
**Capecitabine**	0.53	0.88	0.9	0.42	0.66	0.93	0.87	0.74	0.75	0.79
**Vinorelbine**	0.25	0.69	0.42	0.16	0.02	0.02	0.58	0.04	0.21	0.65
**Paclitaxel**	0.57	0.04	0.08	0.23	0.13	0.06	0.57	0.01	0.05	0.02
**Leucovorin**	0.26	1.00	0.53	0.27	0.34	0.71	0.48	0.12	0.60	0.36
**Cyclophosphamide**	0.66	0.34	0.39	0.6	0.23	0.03	0.96	0.51	0.69	0.24
**Methotrexate**	0.09	0.14	0.14	0.14	0.98	0.83	0.09	0.43	0.27	0.12
**Dacarbazine**	0.31	0.45	0.82	0.15	0.24	0.97	0.52	0.20	0.45	0.86
**Ifosfamide**	0.03	0.28	0.54	0.01	0.87	0.06	0.14	0.22	0.16	0.39
**Gemcitabine**	0.53	0.03	0.74	0.10	0.13	0.61	0.19	0.02	0.06	0.03
**Tamoxifen**	0.34	0.16	0.12	0.99	0.99	0.39	0.59	0.05	0.13	0.02
**Vincristine**	0.04	0.58	0.12	0.58	0.85	0.53	0.02	0.05	0.07	0.93
**Irinotecan**	0.04	0.53	0.76	0.19	0.89	0.81	0.40	0.71	0.81	0.66
**Cisplatin**	0.03	0.02	0.00	0.01	0.03	0.91	0.22	0.02	0.06	0.18
**Oxaliplatin**	0.36	0.33	–	0.86	0.33	0.95	0.22	0.09	0.03	0.06
**Carboplatin**	0.04	0.12	–	0.09	0.71	0.83	0.01	0.55	0.73	0.69
**Vinblastine**	0.73	0.82	0.66	0.62	0.18	0.27	0.30	0.80	0.50	0.34

Table S3. The results of cross-dataset analysis over different feature reduction methods using elastic net regression. Shown are p-values of a one-sided Mann-Whitney-Wilcoxon test for association between DRP prediction and ground truth.

	All gene expressions	Landmark genes	Drug pathway genes	OncoKB genes	Pathway activities	TF activities	Highly correlated genes	Top PCs	Top SPCs	Autoencoder embedding
**Doxorubicin**	0.80	0.36	0.19	0.27	0.64	0.65	0.51	0.02	0.94	0.31
**Temozolomide**	0.96	0.21	0.73	0.94	0.51	0.84	0.19	0.66	0.81	0.03
**Docetaxel**	0.34	0.60	0.09	0.12	0.63	0.77	0.42	0.25	0.98	0.23
**Etoposide**	0.12	0.31	0.00	0.01	0.22	0.19	0.11	0.69	0.46	0.94
**Capecitabine**	0.63	0.84	0.91	0.41	0.69	0.94	0.83	0.76	0.76	0.79
**Vinorelbine**	0.22	0.69	0.42	0.42	0.02	0.01	0.78	0.04	0.22	0.69
**Paclitaxel**	0.37	0.03	0.07	0.25	0.13	0.07	0.42	0.01	0.06	0.02
**Leucovorin**	0.50	1.00	0.55	0.55	0.27	0.79	0.44	0.06	0.60	0.32
**Cyclophosphamide**	0.84	0.22	0.54	0.34	0.22	0.03	0.97	0.57	0.72	0.26
**Methotrexate**	0.17	0.17	0.04	0.23	0.97	0.86	0.09	0.48	0.31	0.17
**Dacarbazine**	0.39	0.17	0.72	0.22	0.22	0.96	0.22	0.18	0.43	0.86
**Ifosfamide**	0.01	0.35	0.54	0.01	0.87	0.06	0.02	0.22	0.16	0.35
**Gemcitabine**	0.13	0.01	0.54	0.03	0.14	0.58	0.21	0.02	0.07	0.03
**Tamoxifen**	0.27	0.41	0.27	0.93	1.00	0.36	0.46	0.04	0.13	0.02
**Vincristine**	0.07	0.73	0.31	0.69	0.85	0.47	0.05	0.05	0.07	0.95
**Irinotecan**	0.53	0.71	0.24	0.34	0.89	0.85	0.71	0.66	0.81	0.66
**Cisplatin**	0.01	0.00	0.15	0.00	0.03	0.88	0.30	0.03	0.09	0.18
**Oxaliplatin**	0.27	0.58	–	0.89	0.31	0.94	0.15	0.05	0.03	0.06
**Carboplatin**	0.05	0.04	–	0.15	0.69	0.91	0.01	0.55	0.75	0.67
**Vinblastine**	0.42	0.85	0.70	0.70	0.18	0.18	0.38	0.8	0.46	0.34

Table S4. The results of cross-dataset analysis over different feature reduction methods using random forest regression. Shown are p-values of a one-sided Mann-Whitney-Wilcoxon test for association between DRP prediction and ground truth.

	**All gene expressions**	**Landmark genes**	**Drug pathway genes**	**OncoKB genes**	**Pathway activities**	**TF activities**	**Highly correlated genes**	**Top PCs**	**Top SPCs**	**Autoencoder embedding**
**Doxorubicin**	0.90	0.98	0.89	0.96	0.06	0.99	0.96	0.43	1.00	0.63
**Temozolomide**	0.65	0.58	0.68	0.29	0.18	0.18	0.82	0.99	0.60	0.40
**Docetaxel**	0.83	0.64	0.76	0.44	0.06	0.34	0.89	0.72	1.00	0.66
**Etoposide**	0.00	0.00	0.00	0.00	0.11	0.10	0.00	0.11	0.00	0.15
**Capecitabine**	0.35	0.01	0.41	0.18	0.45	0.03	0.31	0.38	0.43	0.45
**Vinorelbine**	0.27	0.83	0.08	0.35	0.03	0.64	0.55	0.78	0.49	0.94
**Paclitaxel**	0.03	0.00	0.02	0.08	0.48	0.06	0.08	0.45	0.09	0.17
**Leucovorin**	0.38	0.01	0.28	0.79	0.61	0.29	0.71	0.05	0.98	0.40
**Cyclophosphamide**	0.50	0.33	0.16	0.69	0.37	0.65	0.04	0.51	0.15	0.54
**Methotrexate**	0.23	0.35	0.73	0.27	0.77	0.61	0.31	0.04	0.04	0.35
**Dacarbazine**	0.48	0.75	0.80	0.12	0.47	0.03	0.06	0.99	0.95	0.90
**Ifosfamide**	0.16	0.51	0.98	0.26	0.54	0.65	0.08	0.92	0.26	0.46
**Gemcitabine**	0.18	0.26	0.28	0.03	0.72	0.02	0.17	0.12	0.00	0.60
**Tamoxifen**	0.18	0.44	0.59	0.80	0.97	0.46	0.59	0.23	0.54	0.32
**Vincristine**	0.96	0.99	0.99	0.96	0.91	0.93	0.99	0.98	0.98	0.22
**Irinotecan**	0.98	0.98	0.96	0.96	0.40	0.99	0.98	0.34	0.89	0.60
**Cisplatin**	0.25	0.11	0.26	0.05	0.10	0.07	0.03	0.95	0.29	0.89
**Oxaliplatin**	0.20	0.13	–	0.27	0.53	0.85	0.13	0.01	0.12	0.25
**Carboplatin**	0.62	0.79	–	0.88	0.88	0.35	0.57	0.66	0.67	0.67
**Vinblastine**	0.11	0.50	0.15	0.18	0.07	0.01	0.13	0.66	0.27	0.13

Table S5. The results of cross-dataset analysis over different feature reduction methods using support vector machine regression. Shown are p-values of a one-sided Mann-Whitney-Wilcoxon test for association between DRP prediction and ground truth.

	**All gene expressions**	**Landmark genes**	**Drug pathway genes**	**OncoKB genes**	**Pathway activities**	**TF activities**	**Highly correlated genes**	**Top PCs**	**Top SPCs**	**Autoencoder embedding**
**Doxorubicin**	1.00	1.00	0.98	1.00	0.3	0.84	1.00	0.58	1.00	0.98
**Temozolomide**	0.99	0.57	0.53	0.63	0.13	0.98	0.97	0.86	0.86	0.61
**Docetaxel**	0.94	0.93	0.95	0.87	0.58	0.58	0.93	0.36	0.96	0.92
**Etoposide**	0.01	0.01	0.01	0.01	0.22	0.08	0.01	0.27	0.03	0.64
**Capecitabine**	0.79	0.60	0.58	0.88	0.84	0.10	0.48	0.41	0.39	0.75
**Vinorelbine**	0.62	0.75	0.62	0.55	0.02	0.87	0.62	0.15	0.89	0.44
**Paclitaxel**	0.22	0.08	0.18	0.12	0.06	0.03	0.12	0.08	0.12	0.19
**Leucovorin**	0.92	0.33	0.2	0.94	0.68	0.02	0.77	0.48	0.79	0.26
**Cyclophosphamide**	0.28	0.26	0.09	0.56	0.31	0.52	0.24	0.24	0.35	0.58
**Methotrexate**	0.88	0.91	0.96	0.91	0.35	0.65	0.31	0.04	0.99	0.01
**Dacarbazine**	0.57	0.84	0.93	0.34	0.34	0.11	0.58	0.39	0.98	0.99
**Ifosfamide**	0.02	0.01	0.01	0.05	0.56	0.01	0.03	0.28	0.16	0.16
**Gemcitabine**	0.25	0.40	0.25	0.34	0.42	0.22	0.11	0.36	0.26	0.01
**Tamoxifen**	0.05	0.12	0.06	0.27	0.96	0.20	0.54	0.23	0.14	0.11
**Vincristine**	0.91	0.96	0.96	0.93	0.85	0.97	0.95	0.85	0.91	0.98
**Irinotecan**	0.99	0.99	0.99	0.99	0.91	0.76	0.99	0.71	0.99	0.85
**Cisplatin**	0.00	0.00	0.01	0.01	0.47	0.00	0.01	0.17	0.00	0.94
**Oxaliplatin**	0.42	0.31	–	0.56	0.36	0.8	0.32	0.40	0.28	0.31
**Carboplatin**	0.86	0.96	–	0.97	0.09	0.92	0.98	0.62	0.37	0.46
**Vinblastine**	0.02	0.01	0.02	0.01	0.09	0.02	0.02	0.80	0.27	0.50

Table S6. The results of cross-dataset analysis over different feature reduction methods using multilayer perceptron regression. Shown are p-values of a one-sided Mann-Whitney-Wilcoxon test for association between DRP prediction and ground truth.

	All gene expressions	Landmark genes	Drug pathway genes	OncoKB genes	Pathway activities	TF activities	Highly correlated genes	Top PCs	Top SPCs	Autoencoder embedding
**Doxorubicin**	1.00	0.99	0.99	0.99	0.81	0.80	0.98	0.61	0.98	0.99
**Temozolomide**	0.85	0.42	0.12	0.85	0.37	0.94	0.98	0.88	0.73	0.63
**Docetaxel**	0.49	0.86	0.88	0.68	0.99	0.55	0.89	0.70	0.99	0.88
**Etoposide**	0.10	0.01	0.01	0.01	0.56	0.03	0.03	0.14	0.00	0.49
**Capecitabine**	0.78	0.72	0.85	0.54	0.51	0.06	0.57	0.60	0.58	0.76
**Vinorelbine**	0.20	0.60	0.56	0.35	0.09	0.91	0.44	0.08	0.27	0.36
**Paclitaxel**	0.03	0.05	0.03	0.02	0.63	0.02	0.11	0.03	0.09	0.16
**Leucovorin**	0.86	0.36	0.44	0.75	0.55	0.00	0.45	0.53	0.61	0.28
**Cyclophosphamide**	0.17	0.36	0.71	0.56	0.83	0.69	0.42	0.40	0.83	0.58
**Methotrexate**	0.77	0.61	0.03	0.65	1.00	0.52	0.04	0.57	0.48	0.01
**Dacarbazine**	0.15	0.78	0.58	0.36	0.28	0.76	0.31	0.17	0.36	0.99
**Ifosfamide**	0.01	0.03	0.11	0.01	0.33	0.01	0.03	0.02	0.06	0.16
**Gemcitabine**	0.00	0.02	0.22	0.07	0.00	0.06	0.03	0.01	0.03	0.01
**Tamoxifen**	0.51	0.70	0.56	0.27	0.99	0.07	0.54	0.27	0.23	0.12
**Vincristine**	0.69	0.96	0.82	0.85	0.95	0.97	0.73	0.22	0.22	0.97
**Irinotecan**	0.98	0.99	0.99	0.99	0.98	0.94	0.99	0.81	0.94	0.76
**Cisplatin**	0.07	0.00	0.19	0.08	0.00	0.00	0.01	0.06	0.05	0.97
**Oxaliplatin**	0.26	0.26	–	0.38	0.27	0.82	0.26	0.41	0.03	0.29
**Carboplatin**	0.73	0.94	–	0.96	0.84	0.91	0.88	0.81	0.80	0.53
**Vinblastine**	0.07	0.01	0.58	0.04	0.06	0.01	0.07	0.70	0.04	0.58

Table S7. Detailed analysis of ridge regression models that were able to distinguish drug-sensitive from drug-resistant tumors.

**Drug name**	**Transcription factors with highest model coefficients**
Etoposide	E2F7, HES1, XBP1, TFDP1, HNF4G, STAT6, ESRRA, ATF6, CDX2, RARB
Paclitaxel	PROX1, E2F7, HNF1B, ZC3H8, GLI1, CEBPG, XBP1, NRF1, WT1, CEBPB
Leucovorin	WT1, POU2F2, GLI1, SIX2, SOX6, AHR, BCL6, ZBTB11, TFAP4, ELF1
Ifosfamide	SP3, ATF6, E2F3, RFX2, TCF3, BACH1, NR2C2, E2F6, MEIS2, ATF1
Gemcitabine	IRF1, CREB1, TP73, SPIB, FOXP2, MYC, TEAD2, NR1H3, ESRRA, E2F2
Cisplatin	NFE2L1, FOXJ2, HOXB13, E2F3, ATF3, PAX5, KMT2B, FOXP2, CEBPG, SIX2
Vinblastine	PROX1, ZC3H8, ESRRA, NR1H4, RARG, GLI1, STAT6, CEBPG, STAT5B, SOX13
